# Systemic Nickel Allergy Syndrome: A Critical Appraisal of an Unvalidated Diagnostic Entity

**DOI:** 10.1002/clt2.70169

**Published:** 2026-05-18

**Authors:** Antonello Giovannetti

**Affiliations:** ^1^ Department of Translational and Precision Medicine Sapienza University of Rome Rome Italy

## Abstract

Systemic Nickel Allergy Syndrome (SNAS) remains a controversial clinical construct, characterized by persistent diagnostic uncertainty and the absence of reproducible biomarkers. Available studies are predominantly limited to small case series and uncontrolled observational reports, yielding partial and inconclusive findings. Diagnostic approaches, including oral provocation tests, frequently employ nickel exposures that far exceed typical dietary intake, raising concerns regarding physiological relevance and methodological validity. Finally, scientific inquiry into this entity has largely neglected crucial aspects such as nickel bioavailability and bioaccessibility. Against this background, particularly within the Italian clinical context, SNAS has received considerable medical and public attention despite the limited and inconsistent evidence supporting its recognition as a systemic allergic condition. Reported clinical improvements following low‐nickel diets are more plausibly explained by non‐specific dietary modifications—such as reduced intake of fermentable carbohydrates—rather than by a targeted immunological response to nickel. In conclusion, SNAS does not fulfill the criteria required for recognition as a discrete pathological entity. Rather, it represents a unvalidated diagnostic label for non‐specific symptoms shared across established functional syndromes.

## 1 Introduction

The term *Systemic Nickel Allergy Syndrome* (SNAS) was introduced to describe patients with nickel sensitization who present with allergic contact dermatitis (ACD) and extracutaneous symptoms [1, 2]. Originally proposed as an extension of Systemic Contact Dermatitis (SCD), SNAS gradually evolved into a distinct entity progressively diverging from the original SCD concept, with extracutaneous manifestations becoming a central focus. SCD, more accurately termed “systemically reactivated allergic contact dermatitis”, refers to a cutaneous condition in which an individual previously sensitized through the skin develops eczematous skin manifestations at distant sites following systemic exposure to the same allergen or a cross‐reacting substance. Importantly, despite the term “systemic”, SCD remains a dermatological entity characterized by skin involvement and does not imply extracutaneous systemic symptoms.

The diagnosis of SNAS has historically relied on a positive oral provocation test combined with the perceived benefit of a low‐nickel diet. Although initially associated with a wide spectrum of systemic manifestations, the syndrome has gradually shifted toward a clinical picture dominated by gastrointestinal disturbances, while cutaneous features—once central to its definition—have been relegated to a secondary role. More recent reports even describe the diagnosis of SNAS as being based solely on positive patch tests, in the absence of allergic contact dermatitis and without confirmatory provocation testing [3].

SNAS has not been universally accepted as a distinct nosological entity. For a clinical condition to be defined as a disease, several requirements must be met, including consistent clinical evidence, reproducible diagnostic criteria, confirmation through rigorous research, demonstrable response to treatment, and eventual recognition in international classifications. A critical appraisal of the available literature indicates that these criteria are not fully satisfied in the case of SNAS, and that current evidence remains insufficient to support its recognition as a distinct clinical entity [4]. Nevertheless, SNAS has attracted disproportionate media and public attention, in a manner reminiscent of Non‐Celiac Wheat Sensitivity (NCWS) and Idiopathic Environmental Intolerance/Multiple Chemical Sensitivity (IEI/MCS). As in these conditions, the wide range of often subtle and nonspecific symptoms attributed to SNAS facilitates self‐diagnosis and symptom attribution particularly in the absence of validated diagnostic biomarkers.

In summary, despite extensive debate and significant media resonance, SNAS remains poorly defined and lacks convincing scientific validation. A more rigorous and critical appraisal of the available evidence is required before it can be considered as a distinct clinical entity.

## 2 Methods

A search on PubMed Advanced using the following terms: (((systemic) AND (nickel)) AND (allergy)) AND (syndrome) yielded 58 results. Among the eligible studies (*n* = 54), 29 were excluded from our review because they were unrelated to SNAS. Of the remaining 25, 15 were excluded because although referred to SNAS they were reviews, case reports and letters with methodological limitations, or described patients with isolated positive nickel patch tests for whom the attribution of a SNAS diagnosis was not supported by consistent clinical or diagnostic criteria (Table 1).

**TABLE 1:** Studies excluded from the review.

Mori et al. [5]	Case report of diarrhea
Risi et al. [6]	Methodologically limited findings, patch test only
Rizzi, Chini, et al. [3]	Methodologically limited findings, patch test only
Caruso et al. [7]	Methodologically limited findings, patch test only
Nucera et al. [8]	Case report of eosinophilic esophagitis
Ricciardi et al. [9]	Letter with unreliable findings
Rizzi et al. [10]	IBS patients
Andrioli et al. [11]	Letter, not relevant to SNAS (nickel allergy and thyroiditis)
Pizzutelli [12]	Comment
Goldenberg and Jacob [13]	Comment
Randazzo et al. [14]	Patch test only, no low‐nickel diet, no oral challenge
Tammaro et al. [15]	Letter, unreliable findings
Ricciardi et al. [16]	Letter, findings of limited reliability due to major methodological flaws
Cazzato et al. [17]	Letter, findings of limited reliability
Pizzutelli [4]	Review

*Note:* List of articles excluded after full‐text assessment, with reasons for exclusion.

**FIGURE 1:**
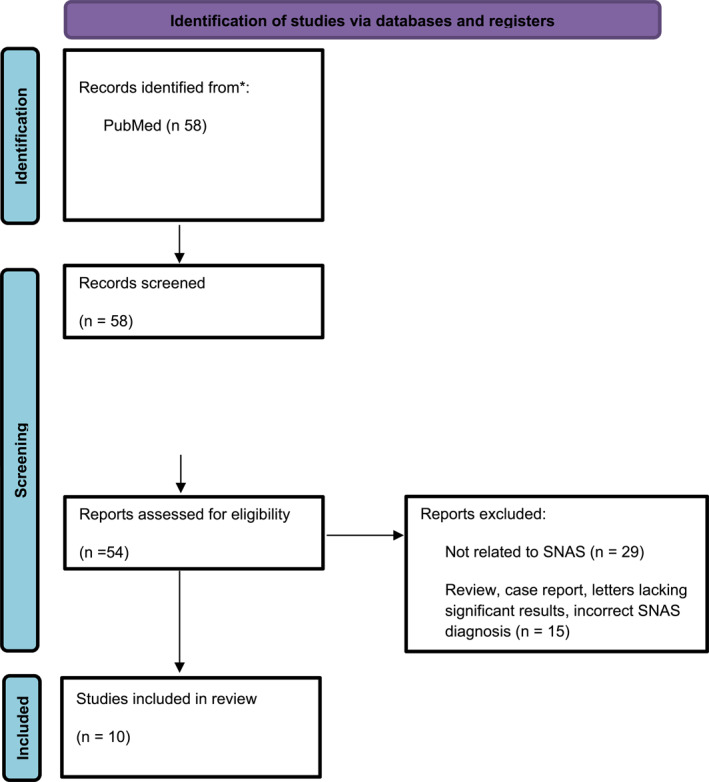
Study selection process.

The study selection process is illustrated in the PRISMA flow diagram (Figure 1).

Therefore, only 10 studies could be considered for the purpose of this review, as they provided data on patients diagnosed with SNAS defined according to diagnostic criteria proposed by the original Italian research groups, including oral challenges, elimination diets, positive patch tests, allergic contact dermatitis, and extracutaneous symptoms (Table 2).

**TABLE 2:** Studies included in the review.

Authors	Purpose	Summary points/major findings	Criticism/weakness
Rizzi et al. [18]	To compare effects of hydroponic versus conventional tomato puree.	Hydroponic tomato puree plus low‐nickel diet led to symptom improvement.	— Inconclusive due to serious methodological weaknesses. — Small sample, 97% female, no control group. — Minimal nickel difference (1–2 μg/100 mL) between the puree. — Patients already on low‐nickel diet expected ≥ 70% improvement. — Paradoxical improvement with hydroponic puree; treatment reversal unexplained.
Nucera et al. [19]	To assess the efficacy of telemedicine tools via remote video calls during the COVID‐19 pandemic.	Telemedicine is valuable for monitoring dietary compliance during remote periods.	— Provides no reliable evidence due to major methodological flaws. — 95% female; 30‐day diet produced minimal changes in weight/BMI; no control group.
Rizzi et al. [20]	Study aimed to assess HRQoL and psychological status after 6 months of oral nickel hyposensitization (NiOH).	Pre‐existing symptoms significantly decreased or disappeared with NiOH therapy (1.5 μg/week for 12 months).	— Low‐quality study; unreliable findings. — Open‐label, 92% female, no control group.
Lombardi et al. [21]	To investigate the effects of probiotics in conjunction with a low‐nickel diet in patients diagnosed with dysbiosis and SNAS.	Probiotics improved dysbiosis versus diet alone.	— Inconclusive due to serious methodological weaknesses. — All had dysbiosis; multiple food restrictions; no controls or nickel absorption assessment; probiotic benefit not novel; low‐nickel diet effectiveness not evaluated before SNAS diagnosis.
Di Gioacchino et al. [22] Schiavino et al. [23] Minelli et al. [24] Ricciardi et al. [25]	To induce tolerance to nickel.	Beneficial effects from oral hyposensitization treatment.	— Low‐quality studies, methodologically inadequate, unreliable findings. — Literature shows contrasting results on nickel tolerance induction; some studies (Sjövall et al. [26] and Morris [27]) report improvement, others (Troost [28], French study) do not. — Italian SNAS groups explored oral nickel therapy, but varied doses and lack of dose‐response make interpretation difficult. — Schiavino et al. [23] reported effects at infinitesimal doses (0.1 ng), raising concerns. — Overall, these studies lacked follow‐up or validation in subsequent trials.
Braga et al. [29]	To assess acceptance and adherence of patients to a low‐nickel diet developed by the author.	Diet proposed by the author is well accepted.	— Inconclusive due to serious methodological weaknesses. — 80% women. Reference (Christensen 1975 [30]) concerns hand eczema, not systemic symptoms
Gangemi et al. [31]	To investigate oxidative stress in patients with nickel allergy.	Slightly higher serum nitrosylated proteins in SNAS versus nickel ACD.	— Major methodological flaws. — 100% women; controls limited (8 vs. 23) and not challenged; single 1.25 mg nickel dose.

*Note:* List of studies meeting the inclusion criteria, with main clinical and methodological characteristics.

## 3 Results

### 3.1 Clinical Features

Early reports suggested that SNAS could involve almost any organ system. Ricciardi et al. described respiratory symptoms (occupational rhinitis, asthma), urticaria, angioedema, gastrointestinal complaints (bloating, abdominal pain, diarrhea/constipation, nausea, vomiting), recurrent aphthous stomatitis, chronic gastroduodenitis, as well as nonspecific systemic manifestations such as headache, chronic fatigue, dyspnea, cystitis, vulvovaginitis, acne, and iron‐deficiency anemia [25]. Subsequent authors proposed a narrower spectrum, restricting diagnostic relevance to cutaneous and gastrointestinal symptoms. Braga et al. noted that manifestations such as headache, dizziness, fatigue, cough, and dyspnea are nonspecific and should not be considered diagnostic for SNAS [29]. These discrepancies highlight the lack of consensus even among its proponents regarding the clinical definition of SNAS. A summary of the methodological and bibliographic shortcomings of frequently cited supportive papers can be found in Table 3.

**TABLE 3:** Methodological and bibliographic shortcomings of studies most often cited in support of SNAS.

Author citing	References cited	Main weaknesses
Braga et al. [29]	Tammaro et al. [15]	Letter, no reliable evidence due to major methodological flaws
	Jensen et al. [32]	A study focused on patients with SCD that do not mention SNAS or extracutaneous disorders throughout the paper.
	“Allergy Vaccine” meeting [33]	Not peer‐reviewed (proceedings); unavailable online
Rizzi et al. [3]	Turi et al. [34]	Misleading reference, claimed JACI, actually *Giornale Italiano di Allergologia e Immunologia Clinica*; not indexed, unavailable.
	Di Gioacchino et al. [35]	*Giornale italiano di medicina del lavoro*, now closed. Not about SNAS; ACD patients
Rizzi [18]	Falagiani [36]	Review, misleading references for SNAS
	Panzani et al. [37]	Not randomized, not controlled, not blinded; dermatitis patients, not SNAS
Di Gioacchino et al. [22]	Di Gioacchino et al. [38]	Self‐citation, SNAS not mentioned; lymphocyte subsets in ACD women
	Gangemi et al. [31]	Not randomized/controlled; women only; no placebo; controls not challenged; nonsignificant results
	Minelli et al. [24]	Self‐citation, poorly designed; limited scientific value
	Jensen et al. [39]	Focus on SCD, not SNAS
	Boscolo et al. [40]	Nickel‐sensitized patients, not SNAS; only 7/19 reacted to very high dose of nickel (10 mg)
Lombardi et al. [21]	Menne and Hjorth [41]	Precedes SNAS term by 27 years; not indexed; unavailable online
Nucera et al. [8]	Brera and Nicolini [42]	20 patients with rhinitis published on Acta ORL Italica, not SNAS; unreliable findings
Minelli et al. [24]	Verna et al. [2]	Short review on nickel allergy; no experimental data on SNAS; not available online.

*Note:* Several of the most frequently cited papers on SNAS are characterized by limited methodological rigor, outdated or inaccessible sources, and frequent self‐referencing. Some consist of brief communications, conference proceedings, or misattributed references rather than original peer‐reviewed research.

### 3.2 Diagnostic Approaches Proposed for SNAS

Several diagnostic approaches have been proposed for SNAS, although none has been formally validated or standardized. The diagnosis has variably relied on combinations of patch testing, oral provocation tests, and reported clinical response to dietary nickel restriction [4].

Patch testing with nickel sulfate identifies delayed‐type hypersensitivity to nickel but does not establish systemic clinical relevance. Positive patch tests are common in the general population and must always be interpreted within an appropriate clinical context. Nevertheless, in several SNAS reports, a positive patch test alone has been considered sufficient for diagnosis, even in the absence of allergic contact dermatitis or confirmatory oral challenge testing.

Oral provocation tests have been regarded by some authors as a key diagnostic criterion. However, these protocols are highly heterogeneous, differing in nickel formulation, administered dose, fasting conditions, and outcome assessment. In most studies, nickel sulfate hexahydrate—a highly soluble synthetic salt—was used to maximize gastrointestinal absorption. Clinical responses were primarily based on patient‐reported symptoms rather than objective or blinded endpoints, limiting reproducibility and interpretability.

Overall, the absence of standardized diagnostic criteria and the reliance on heterogeneous, largely subjective diagnostic approaches represent major limitations in the current SNAS literature.

### 3.3 Challenge With Scientific Research

To date, evidence for SNAS remains limited and methodologically weak. The available studies lack controls, standardization, or validated diagnostic criteria, and no large clinical trials or multicenter studies have been performed. Notably, the syndrome has never been substantiated outside Italy (Table 1). Pizzutelli first pointed out that virtually all publications originate from Italian groups and are concentrated in a small number of mostly local journals [4, 12]. It is striking that a supposedly widespread and disabling condition has gained no international recognition over such a long period.

SNAS lacks robust bibliographic support, as the literature is characterized by self‐referential citations, methodological flaws, and sources that are often inaccessible, outdated, or of limited scientific value. Several frequently cited articles are brief letters, meeting proceedings, or even erroneously attributed to higher‐impact journals (see Table 3). References to non‐Italian studies are often inappropriate, such as Jensen et al. on systemic contact dermatitis [32], Mills et al. on food allergy epidemiology [43], or Erdmann and Werfel's critical review of dietary nickel in contact dermatitis [44]. This pattern underscores the lack of a robust scientific foundation.

### 3.4 Evidence Base and Methodological Concerns

No standardized tests reliably link reported systemic symptoms to nickel exposure, and a correlation between self‐reported complaints and objective measures of physiological dysfunction has never been demonstrated.

Patch testing identifies sensitization to allergens that elicit delayed‐type hypersensitivity in the skin, yet its clinical relevance must always be evaluated given the high prevalence of positive tests among healthy individuals. Similarly, elimination diets are equally problematic because of the ubiquity and variability of dietary nickel and because dietary restriction may overlap with other interventions that improve nonspecific gastrointestinal symptoms.

In SCD, oral provocation studies have yielded inconsistent results. High doses of nickel sulfate administered under fasting conditions have induced flares in a minority of highly sensitized individuals [45, 46], a result not replicated in a later study where outpatients with nickel monosensitization were challenged with even higher doses [47], highlighting the lack of a clear dose–response relationship and further limiting the interpretability of oral provocation results.

Subsequent investigations concluded that the nickel content in a typical diet did not significantly influence the chronicity of dermatitis [48] suggesting that dietary nickel content alone is unlikely to be a reliable determinant of clinical outcomes.

It should be noted that absorption from nickel sulfate hexahydrate used in oral exposure studies is not comparable to absorption of naturally occurring dietary nickel. Nickel sulfate hexahydrate is a synthetic salt used in experimental settings precisely because it is highly soluble [49]. In contrast, in food, nickel is not fully bioaccessible; it is present as the divalent cation Ni2+ bound to complex molecules, and systemic absorption is estimated at 1%–5% [50], limiting the extrapolation of oral challenge results to physiological dietary exposure.

In SNAS studies, a positive oral challenge is considered essential for diagnosis. Methodologies, however, are rarely detailed, and when they are, significant differences emerge [20, 21, 22, 25]. The design of these studies—with subjective endpoints, absence of control and reliance on patient‐reported discomfort—may help explain the near‐universal positivity and sharply contrasts with SCD literature, where flare‐ups were evaluated objectively using SCORAD scores and control groups [46]. Taken together, all these methodological flaws render oral challenge studies unsuitable for clinical practice.

### 3.5 Treatment Issues and Confounding Factors

In routine clinical practice, particularly in Italy, a low‐nickel diet is commonly recommended for patients labeled with SNAS and is often reported to alleviate symptoms. This approach has become so widespread that patients frequently receive extensive lists of foods to avoid based solely on a positive nickel patch test.

These lists, often originating from specific local protocols, mandate the exclusion of a wide array of healthy staples, including tomatoes, legumes, and whole grains. However, it must be emphasized that such extreme restrictions are based on the same methodologically weak studies discussed above [7, 10, 19, 21, 29, 51], rather than on official guidelines from international allergy or nutrition societies. The act of prescribing restrictive diets and providing detailed list of “forbidden” foods may itself influence symptom perception through expectancy and nocebo mechanisms, particularly in conditions characterized by nonspecific and subjective endpoints. This effect may be further amplified by public‐facing medical information and regulatory communications on dietary nickel content, which, although intended for population‐level food safety, can reinforce illness attribution and symptom vigilance at the individual level (EFSA and European Commission) [52]. In this context, the apparent symptomatic benefit reported with low‐nickel diets may reflect contextual and behavioral factors rather than a specific effect of dietary nickel avoidance, further complicating the interpretation of therapeutic responses in patients diagnosed with SNAS. Beyond contextual and expectancy‐related effects, an additional confounding factor is that so‐called low nickel diets substantially overlap with other restrictive dietary patterns known to improve gastrointestinal symptoms. It has been proposed that low‐nickel diets may share functional similarities with low‐FODMAP diets (Fermentable Oligo‐, Di‐, and Monosaccharides and Polyols) considered the gold standard for managing IBS [51]. Notably, low‐FODMAP diets are effective across a broad range of conditions, in addition to irritable bowel syndrome (IBS), gastroesophageal reflux disease (GERD), non‐celiac wheat sensitivity (NCWS), functional dyspepsia, fibromyalgia, quiescent inflammatory bowel disease, and small intestinal bacterial overgrowth (SIBO) [53, 54, 55, 56]. Symptoms such as bloating, the most commonly reported in SNAS, are nonspecific and highly prevalent in IBS, lactose intolerance, and GERD. Nickel sensitivity was significantly higher in IBS patients than in healthy controls suggesting that at least a subset of patients diagnosed with SNAS may have underlying IBS [57]. In a study on GERD patients, a low‐nickel diet was able to reduce GERD‐HRQL, regurgitation, and heartburn scores after 8 weeks. However, responses were nonspecific, as patients with positive and negative patch tests for nickel improved similarly [58]. GERD symptoms improve not only with a low‐nickel diet but also with other interventions, including elimination diets based on testing, probiotic yogurt, psyllium, supplements, melatonin, amino acids, B‐group vitamins, and ginger [59].

Low‐nickel diets have also been associated with symptom relief in endometriosis (dysmenorrhea, dyspareunia, pelvic pain) [60] and reductions in BMI and waist circumference in overweight patients [61]. These observations suggest that the overall health benefits of a so‐called low‐nickel diet arise from general reductions in negative dietary factors, rather than from nickel content or allergic sensitization.

In contrast, dietary interventions do not appear to influence ACD, which is driven by specific T cell‐mediated responses. In a systematic review of food avoidance diets for dermatitis [62] the only study reaching Level II evidence (moderate quality) found no improvement in dermatitis with a low‐nickel diet. Lower‐quality studies showed inconsistent outcomes, and oral food challenges did not consistently correlate with diet response [62]. In a randomized study on patients with eczema and metal allergies participants were assigned to a dietary intervention or control group [63]. Despite lower nickel intake in the control group, symptom improvement was more pronounced in the dietary intervention group suggesting that factors beyond nickel content, including placebo effects, operator influence, and reduction of other symptom‐triggering foods, may drive reported benefits.

### 3.6 Biochemical Inconsistencies

Nickel absorption from food is low (approximately 3%) and depends on bioaccessibility and bioavailability, which are rarely considered in studies estimating nickel exposure and in SNAS studies. The bioaccessible fraction corresponds to the soluble amount of nickel released from the food matrix into digestive fluids during digestion—the maximum potentially absorbable amount. Bioavailability, instead, refers to the fraction that actually crosses the intestinal epithelium and enters the bloodstream [64]. These fractions represent the most relevant determinants of health risk and must be considered in exposure studies. Oral challenges with nickel sulfate under fasting conditions expose patients to doses far exceeding physiological intake—up to 1000‐fold higher—making findings irrelevant to dietary exposure [49], and raise additional safety and interpretative concerns. This undermines the rationale for extrapolating results to everyday nutrition. When viewed through this lens, the values used to define so‐called low‐nickel diets appear largely arbitrary.

### 3.7 Comparisons to Other Conditions

SNAS exhibits substantial symptomatic convergence with idiopathic conditions such as NCWS, IBS, and IEI/MCS. This overlap, together with the absence of definitive organic biomarkers, suggests that these diagnostic labels may function, at least in part, as symptomatic constructs rather than as distinct toxico‐immunological disease entities. NCWS represents a paradigmatic example: even under double‐blind, placebo‐controlled challenges, only a minority of self‐identified patients exhibit reproducible reactions to gluten, while the majority are clinically indistinguishable from IBS [65].

IBS itself is defined by the Rome IV criteria as a disorder of gut–brain interaction rather than a structural or biochemical disease, relying primarily on subjective symptom patterns in the absence of validated biological markers [66]. Similarly, IEI/MCS lacks recognition as an organic disease by major international health organizations, as the attribution of multisystem symptoms to sub‐toxic exposures involving chemically unrelated substances conflicts with established toxicological principles [67]. The clinical management of these conditions is frequently characterized by high rates of self‐prescribed exclusion diets (up to 57.7% in IEI/MCS patients), which lack a validated toxicological basis and may lead to severe complications such as sarcopenia, frailty, and micronutrient deficiencies [68]. These dietary interventions are not necessarily benign and may lack a favorable risk‐benefit ratio. For instance, unnecessary adherence to a gluten‐free diet has been associated with higher blood and urine concentrations of heavy metals (e.g., arsenic and mercury) [69], likely due to rice‐based substitutes, as well as an increased risk of metabolic syndrome [70, 71]. Similarly, prolonged low‐FODMAP diets have been shown to induce potentially harmful alterations in gut microbiota composition, including reductions in beneficial Bifidobacteria and overall microbial diversity [72]. Across these conditions, including SNAS, the reliance on subjective symptom reporting, combined with expectancy and nocebo effects, highlights significant conceptual fragility in current diagnostic frameworks.

### 3.8 Potential Risks of SNAS Diagnosis

Restrictive dietary interventions are frequently portrayed as harmless or even salutary. In reality, they may be associated with potential adverse effects, as documented for other restrictive elimination diets [73]. The exclusion of broad food categories may predispose patients to nutritional deficiencies, particularly in essential micronutrients derived from vegetables. Concerns have been documented regarding low‐FODMAP regimens—specifically nutritional inadequacy [74, 75], altered gut microbiota [76], and disordered eating, while unjustified gluten‐free diets have been linked to increased risk of metabolic syndrome and substantial financial costs [73, 77]. In this context, the low‐nickel diet represents an additional restrictive dietary approach that eliminates a wide range of foods in the absence of robust evidence of benefit, posing similar risks of nutritional inadequacy. In fact, while there is no evidence that dietary nickel restriction confers health benefit—and a completely nickel‐free diet remains practically unachievable—high exposure can indeed be toxic. Although some studies in mammals suggest potential physiological roles of nickel [78, 79], acute or chronic administration in animal models has been associated with diverse outcomes. Evidence from experimental animal models demonstrates that nickel salts, when administered systemically at elevated doses, can elicit multi‐organ toxicity, including reproductive impairment, renal and hepatic injury, neurobehavioral disturbances, and developmental effects [80, 81, 82].

The risks of a canonical SNAS diagnosis extend beyond nutrition. Oral provocation protocols involve the administration of nickel salts at pharmacological rather than dietary doses, often under fasting conditions, thereby maximizing absorption. According to the EFSA Panel of Contaminants in the Food Chain, the tolerable daily intake (TDI) of nickel is 0.9 mg/day for a 70‐kg adult, while the lowest observed adverse effect level (LOAEL) is 0.3 mg/day (based on the elicitation of systemic contact dermatitis) [81]. When compared with the EFSA health‐based guidance values, the amounts of nickel administered in oral challenge protocols fall within, or exceed, ranges associated with adverse effects in experimental models, thereby providing little or no margin of safety. Consequently, these protocols cannot be considered physiologically neutral and raise relevant toxicological and ethical concerns, particularly in the absence of standardized methodologies and robust evidence of diagnostic benefit.

Equally relevant are the psychological consequences. Patients are often informed that nickel is ubiquitous in foods, water, and utensils, making avoidance unattainable. Such information may contribute to heightened anxiety, emotional distress, and social withdrawal, with detrimental effects on quality of life [20].

Finally, the possibility of misdiagnosis represents a major concern. Attributing symptoms to SNAS without adequate exclusion of alternative etiologies—such as inflammatory bowel diseases, GERD, or malignancies—risks delaying appropriate diagnostic work‐up and evidence‐based therapy. From this perspective, the apparent simplicity of diagnosing SNAS may mask considerable clinical and scientific limitations. The principal factors undermining the validity of SNAS as a systemic entity are summarized in Table 4.

**TABLE 4:** Main reasons undermining the credibility of Systemic Nickel Allergy Syndrome (SNAS).

#	Reason	Explanation
1	Lack of standardized diagnostic criteria	Different studies use heterogeneous definitions (patch test alone, oral challenge, dietary response), preventing reproducibility.
2	Methodological weakness of available studies	Evidence derives mainly from case series, uncontrolled observational studies, or anecdotal reports.
3	Nonspecificity of reported symptoms	Gastrointestinal discomfort, fatigue, and diffuse complaints overlap with common disorders (e.g., IBS, food intolerances).
4	Absence of validated biomarkers	No laboratory marker reliably identifies SNAS or predicts clinical response to nickel restriction.
5	Inconsistency of oral provocation tests	Provocation protocols lack standardization, with highly variable and often contradictory results.
6	Lack of mechanistic plausibility	No convincing immunological or pathophysiological evidence supports systemic effects of dietary nickel in sensitized patients.
7	Overemphasis on dietary interventions	Reported benefits of low‐nickel diets are confounded by broad food exclusions, placebo effects, and lifestyle changes.
8	Potential harm of restrictive diets	Low‐nickel regimens can induce nutritional deficiencies, alter gut microbiota, and increase psychological burden.
9	Absence of international recognition	SNAS is not listed in major allergy or dermatology classification systems.
10	Disproportionate media attention	Public visibility exceeds the scientific evidence, fostering misconceptions and diagnostic confusion.

## 4 Conclusion

Systemic Nickel Allergy Syndrome (SNAS) remains a controversial clinical construct characterized by persistent diagnostic uncertainty and the absence of reproducible objective criteria. The available literature is largely composed of small case series and uncontrolled observational studies with significant methodological limitations, while diagnostic definitions remain inconsistent across publications. As a result, the proposed clinical phenotype lacks the internal coherence required for reliable clinical standardization.

From a mechanistic perspective, no convincing evidence demonstrates that dietary nickel consistently induces systemic symptoms in sensitized individuals under physiologically relevant conditions. Oral provocation protocols vary substantially and do not provide a robust or reproducible diagnostic foundation. In parallel, reported clinical improvements following low‐nickel diets are more plausibly explained by non‐specific dietary modifications—such as reduced intake of fermentable carbohydrates—rather than by a clearly established immunological response to nickel. Given these methodological and conceptual limitations, the extensive medical and media attention surrounding SNAS in certain regional contexts—particularly in Italy—is not justified.

Taken together, current data do not support the recognition of SNAS as a validated nosological entity. Rather than representing a distinct systemic allergic disorder, the label appears to aggregate non‐specific symptoms that overlap extensively with established functional conditions. Accordingly, further attempts to formalize SNAS as a discrete systemic disease should be approached with caution. Clinical practice is better served by prioritizing the evidence‐based management of established disorders while maintaining methodological rigor in allergy research.

## Funding

The author has nothing to report.

## Ethics Statement

The author has nothing to report.

## Consent

The author has nothing to report.

## Conflicts of Interest

The author declares no conflicts of interest.

## Data Availability

Data sharing is not applicable to this article, as no new data were generated or analysed during the current study. The review is based entirely on previously published studies available from public sources such as PubMed and journal websites.
